# Deep head and neck infection causing pathological fracture of the mandibular condyle

**DOI:** 10.1259/bjrcr.20160093

**Published:** 2017-01-12

**Authors:** Mette Werner Linderup, Sidse Bregendahl, Malene Helleberg, Sten Schytte, Arunas Pikelis, Sven Erik Nørholt

**Affiliations:** ^1^Department of Oral and Maxillofacial Surgery, Aarhus University Hospital, Aarhus, Denmark; ^2^Department of Neuroradiology, Aarhus University Hospital, Aarhus, Denmark; ^3^Department of Otolaryngology, Aarhus University Hospital, Aarhus, Denmark; ^4^Section of Maxillofacial Surgery and Oral Pathology, Aarhus School of Dentistry, Aarhus University, Aarhus, Denmark

## Abstract

We report an unusual case of a 46-year-old male with a severe deep head and neck infection after extraction of two mandibular molars that led to a potentially life-threatening condition and caused pathological fracture of the mandibular condyle. This is the first published spontaneous pathological fracture of the mandibular condyle caused by an infection spread from a lower molar tooth. Based on CT scan we discuss the pathways of infection of odontogenic origin and the reflections of treatment. This case report illustrates an unusual presentation of a spontaneous pathological condylar fracture caused by a severe life-threatening infection after tooth extraction. It details the examination and management of the patient and reflections about the treatment.

Abscesses of odontogenic origin are common, but in developed countries they rarely progress to life-threatening deep head and neck infections.^1^ When extended abscess formation is seen, it is not adequate to treat with orally administered antibiotics. Deep head and neck infections with an odontogenic origin require an immediate and accurate treatment. Elimination of the source of infection, drainage and administration of intravenous antibiotics is the treatment of choice.^2^ Insufficiently treated odontogenic abscesses can spread to a life-threatening infection in the retropharyngeal space and have a high risk of caudal extension.^1^ Furthermore, infections of odontogenic origin often cause osteomyelitis of the jaw in relation to the infected tooth, which results in a risk of spontaneous pathological fracture.^3^

## Case report

### Patient history and clinical findings

A 46-year-old male was referred to the Department of Oral and Maxillofacial Surgery, Aarhus University Hospital, Denmark, with a 3-week history of infection after tooth extraction in the left side of the mandible. He was diagnosed with gout in 2004, and was on regular medication with allopurinol, prednisolone and alendronate for the last 14 months. He was a non-smoker with moderate use of alcohol (two to three units daily).

**Figure 1.**
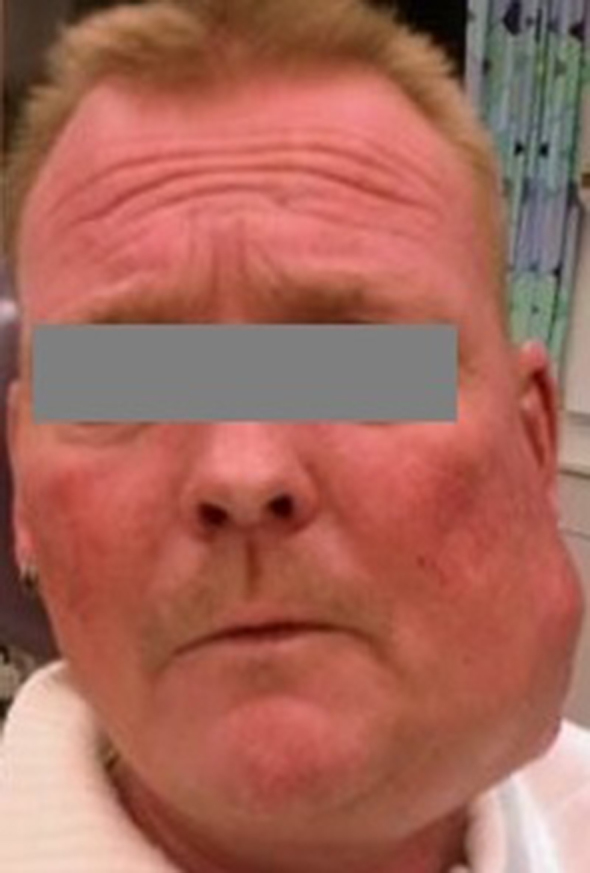
Preoperative photo demonstrating severe swelling of the left side of the face.

**Figure 2.**
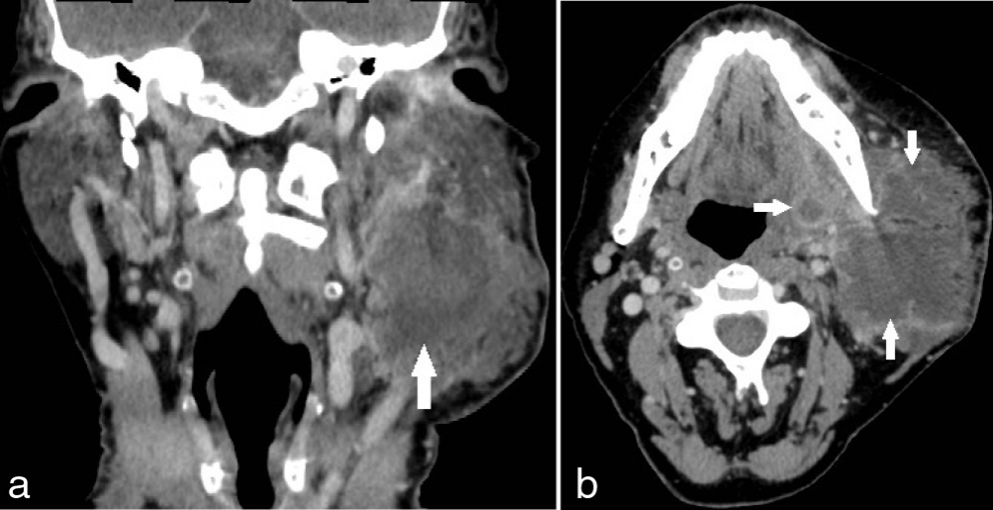
Preoperative venous phase contrast-enhanced CT images showing left side soft tissue enlargement of the face and neck with abscess cavities in (a) coronal reformat and (b) axial reformat.

**Figure 3.**
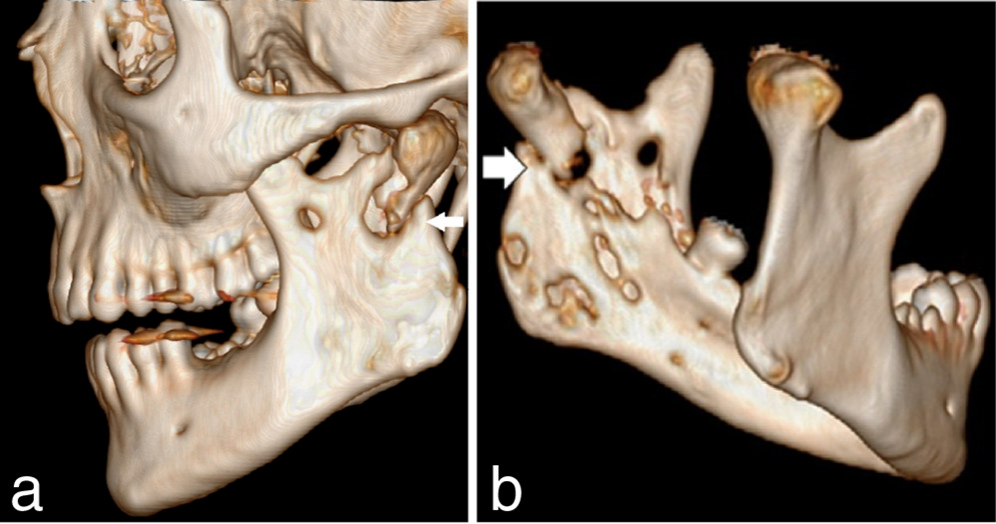
Preoperative 3D-CT volume-rendered reconstructed images demonstrating osteomyelitis and a pathological fracture of the mandibular condyle, which is marked with arrows.

The patient presented with a large swelling in the left side of the face with pus from the extraction sockets of the lower left first and second molars. Despite medical advice he refused admission for treatment with intravenous antibiotics and drainage, but accepted a prescription for oral antibiotics (penicillin one MIU four times daily and metronidazol 500 mg three times daily) to use at home. One month later, he went to his general dentist with persistent infection and worsening of the swelling. The infection had escalated dramatically and he was immediately admitted to the hospital for treatment. Subjective symptoms were trismus, moderate pain and the feeling of impending rupture. He felt no impairment of breathing or pain when swallowing, but he had been sleeping in a sitting position and only had liquid food for several days. Objectively, a large swelling on the left side of the face extended from the zygomatic arch to the submandibular region and with a focus of the size of a tennis-ball around the mandibular angle. The swelling was warm, red and had several non-ruptured pus-filled foci (Figure 1). His body temperature was 37.7 °C, C-reactive protein was 125 mg l^–1^, white cell count was 13800 µl and blood pressure 108/68 mmHg with a pulse rate of 97. Intraorally, there were signs after extraction of two molars of the left side of the mandible, no spontaneous pus from the alveolus and only a slight swelling of the floor of the mouth on the left side. The patient proceeded to venous phase contrast-enhanced CT imaging, which was performed using a dual-source 128-multiple detector CT scanner (Somatom Definition Flash, Siemens Healthcare). CT imaging demonstrated a transspatial, multilocular abscess with extension from the zygomatic arch to the level of C4/C5 and posterior from the sternocleidomastoid muscle along the base of the left mandible to the midline involving the buccinator space, the masticator space, the submandibular space, the lateral pharyngeal space and the retropharyngeal space (Figure 2a,b). Bone resorption at the lingual aspect of the molar region in the left mandible indicated the origin of infection from a molar tooth. The extension of the infection both on the lateral and the medial aspects of the left condyle led to bone destruction and fracture with some displacement of the left mandibular condyle (Figure 3a,b). Diffuse stranding, reduced space in oropharynx, compression of the internal jugular vein and several enlarged lymphatic glands of the left side of the neck were observed.

## Treatment and follow-up

**Figure 4.**
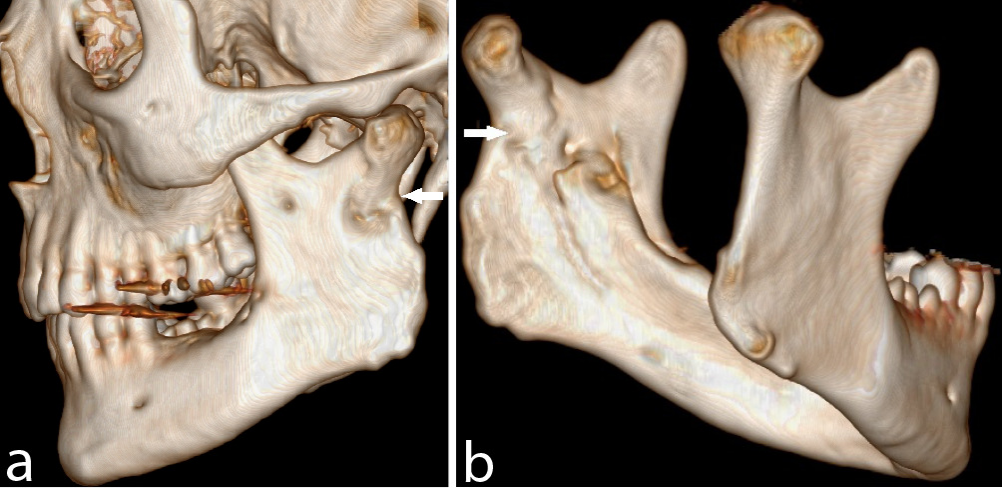
Six-months postoperative 3D-CT volume-rendered reconstructed images demonstrating healing of the condylar fracture in a slightly shortened and anteriorly displaced position. Arrows mark the healed fracture.

The patient was admitted to the hospital in the evening and was initially given intravenous antibiotics (1.2 g benzylpenicillin and 1000 mg metronidazol daily) overnight. In the early morning, before surgery, the abscess ruptured spontaneously with drainage of a large amount of pus. The patient had a nasoendotracheal tube by use of fiberoptic technique. The surgical approach consisted of extraction of the left lower third molar, drainage of around 200 ml of pus, debridement by intra- and extraoral approach, biopsy of soft tissue and bone and insertion of drainage catheter intra- and extra orally. Abscess specimens were submitted to microbiological examination. Because of swelling in the airways, the patient was sedated and kept intubated for 48 h in the intensive care unit where the antibiotic treatment regime was changed to intravenous piperacillin/tazobactam (4 g 0.5 g^–1^ three times daily). The duration of the stay at the hospital was 6 days, after which he was discharged with per oral antibiotics (amoxicillin + clavulanic acid (500 mg + 125 mg) and metronidazol (500 mg) three times daily). Two days after discharge he was rehospitalized to have an acute second surgery for supplementary drainage and another 4 days with intravenous antibiotics ((cefuroxim 1500 mg three times daily) and metronidazol (500 mg two times daily)). He finished his treatment with oral antibiotics for 7 days (amoxicillin + clavulanic acid and metronidazol). Bacterial cultures returned positive with coagulase-negative staphylococcus sensitive to penicillin. Biopsies revealed no signs of malignancy and showed inflammation and necrotic bone. The pathological fracture of the mandibular condyle was managed non-surgically and a 4-month clinical control showed a good mandibular mobility, normal occlusion and good function with a slight deviation to the left at the maximal jaw opening. CT after 5 months illustrated good healing of the bone destruction (Figure 4a,b). The condyle fracture had healed with some shortening of the condylar neck, and a slightly anterior position of the condylar head in the mandibular fossa. There was total regression of the abscess and no contrast enhancing processes.

## Discussion

We present a patient with a pathological fracture of the mandibular condyle caused by a deep head and neck infection spread after extraction of lower molar teeth. A large number of cases and retrospective studies have shown that a pathological fracture can be a complication to an odontogenic abscess, but spontaneous pathological fractures most often appear in the body and angle region of the mandible.^4,5^ Spontaneous pathological fractures of the condyle have mostly been reported to be caused by osteoradionecrosis, tumours and bisphosphonate-related osteonecrosis.^3^ The use of bisphosphonate causes a risk of osteonecrosis of the jaws and the risk for pathological fracture.^6,7^ In the present case, alendronate—a low-dose bisphosphonate—had been administered for approximately 1 year in combination with prednisolone. Alendronate and prednisolone in combination might act synergistically and pose a higher risk of osteonecrosis, but it will remain unclear whether it had any influence on the fracture in this case.^8^ The authors believe that the main reason for the pathological fracture of the condyle was the extensive period (6 to 8 weeks) with severe infection. The oral cavity contains a rich and abundant microflora and more than 250 bacterial strains can be isolated from head and neck space infections of odontogenic origin.^9^ In our patient, coagulase-negative staphylococci were cultured from the pus and those are of the most common bacteria found in cultures from submandibular abscesses.^10^ Boscolo-Rizzo and Da Misto retrospectively reviewed 81 patients with submandibular abscesses.^11^ Of these, 42 patients had a microbiological diagnosis, and coagulase- negative staphylococci were present in 38.1%.^6^ In contrast to these findings, Warnke et al found viridans streptococci to be the most dominant aerobic species in odontogenic abscesses (54%), and only found coagulase-negative staphylococcus in 5.7% of the pus-samples from 94 patients.^12^ In the anaerobic group, the prevotella species dominated (52.7%). This study also compared the *in vitro* sensibility of the present bacteria to penicillin, amoxicillin with clavulanic acid, doxycycline, clindamycin and moxifloxacin, and demonstrated that moxifloxacin had the highest (87%) and penicillin the lowest (20%) *in vitro* efficacy against bacteria species isolated from each patient. But in the era of trying to minimize the use of broad spectrum antibiotics to avoid multiresistant species, they investigated the clinical effect of the different antibiotics. There was very good clinical success with the use of penicillin because of the high susceptibility to penicillin of the dominant strains found and they recommend that penicillin should still be the first choice in patients with severe odontogenic abscesses.^12^ A study by Poeschl et al^2^ in 2010 found the same dominant strains and they used amoxicillin with clavulanic acid as their first choice without any clinical failure of the treatment. In our patient, the type of antibiotics administered was changed from penicillin to broad-spectrum types (piperacillin/tazobactam), which was influenced by his condition with compromised airways and the need for intensive care. He was also treated surgically with incision and drainage, which is one of the oldest treatments in medical history and still the treatment of choice often without the need for antibiotics.^12^ Management of condylar fractures can be surgical or conservative.^13,14^ Owing to the pathological spontaneous origin of the fracture a non-surgical approach was our first choice and showed a good result. A deep head and neck infection is potentially lethal owing to the risk of airway obstruction and spread of the infection to the mediastinum. Infections from the apical aspect of the teeth are prone to penetrate where the bone is thinnest. In the second and third molar region of the mandible, an odontogenic infection will most often penetrate at the lingual side extending into the submandibular space. From here, the infection can diffuse through contiguous spaces. Once the infection reaches the retropharyngeal space, the infection may extend downwards into the mediastinum with the risk of causing mediastinitis and necrotizing fasciitis.^15^ In our case, CT indicated a pan-space infection after extraction of two mandibular molars, including the buccal, submandibular, masticator, lateral pharyngeal and upper retropharyngeal spaces with osseous destruction of the mandible and a pathological fracture.

## Learning points

1. Patients with long-standing, untreated head and neck infections and potentially lethal progression are rare, and many physicians will have limited experience in dealing with the complications. In developed countries, there is easy access to health care and treatment with antibiotics, resulting in a short time delay from diagnosis to treatment. However, patients should be informed that they should seek the doctor again for reassessment if an escalating infection does not respond to per oral antibiotics.
2. The standard approach to deep head and neck infections of odontogenic origin is IV antibiotics, surgical debridement and drainage. This was also chosen in this case with a good result.
3. 3D radiological imaging can give valuable information regarding infection foci and fractures compared to conventional 2D X-ray.

## Consent

Informed consent for the case to be published (including images, case history and data) was obtained from the patient.

## References

[1] Reynolds SC, Chow AW. Life-threatening infections of the peripharyngeal and deep fascial spaces of the head and neck. Infect Dis Clin North Am 2007; 21: 557–76. 17561083 10.1016/j.idc.2007.03.002

[2] Poeschl PW, Spusta L, Russmueller G, Seemann R, Hirschl A, Poeschl E, et al Antibiotic susceptibility and resistance of the odontogenic microbiological spectrum and its clinical impact on severe deep space head and neck infections. Oral Surg Oral Med Oral Pathol Oral Radiol Endod 2010; 110: 151–6. 20346713 10.1016/j.tripleo.2009.12.039

[3] Carlsen A, Marcussen M. Spontaneous fractures of the mandible concept and treatment strategy. Medicina Oral Patol Oral y Cir Bucal 2016; 21: e88–94. 10.4317/medoral.20716 PMC4765750 26636905

[4] Boffano P, Roccia F, Gallesio C, Berrone S. Pathological mandibular fractures: a review of the literature of the last two decades. Dent Traumatol 2013; 29: 185–96. 23294978 10.1111/edt.12028

[5] Gerhards F, Kuffner H-D, Wagner W. Pathological fractures of the mandible. Int J Oral Maxillofac Surg 1998; 27: 186–90. 9662010 10.1016/s0901-5027(98)80007-6

[6] Boscolo-Rizzo P, Da Mosto MC. Submandibular space infection: a potentially lethal infection. Int J Infect Dis 2009; 13: 327–33. 18952475 10.1016/j.ijid.2008.07.007

[7] Chrcanovic BR. Surgical versus non-surgical treatment of mandibular condylar fractures: a meta-analysis. Int J Oral Maxillofac Surg 2015; 44: 158–79. 25457827 10.1016/j.ijom.2014.09.024

[8] van den Bergh B, Blankestijn J, van der Ploeg T, Tuinzing DB, Forouzanfar T Conservative treatment of a mandibular condyle fracture: comparing intermaxillary fixation with screws or arch bar. A randomised clinical trial. J Craniomaxillofac Surg 2015; 43: 671–6. 25911121 10.1016/j.jcms.2015.03.010

[9] Rana RS, Moonis G. Head and neck infection and inflammation. Radiol Clin North Am 2011; 49: 165–82. 21111134 10.1016/j.rcl.2010.07.013

[10] Otto S, Pautke C, Hafner S, Hesse R, Reichardt LF, Mast G, et al Pathologic fractures in bisphosphonate-related osteonecrosis of the jaw-review of the literature and review of our own cases. Craniomaxillofac Trauma Reconstr 2013; 6: 147–54. 24436752 10.1055/s-0033-1343776 PMC3773037

[11] Wongchuensoontorn C, Liebehenschel N, Wagner K, Fakler O, Gutwald R, Schmelzeisen R, et al Pathological fractures in patients caused by bisphosphonate-related osteonecrosis of the jaws: report of 3 cases. J Oral Maxillofac Surg 2009; 67: 1311–6. 19446222 10.1016/j.joms.2008.12.030

[12] Reiss S, Sultan D. Risk factors in the development of oral bisphosphonate-induced osteonecrosis. N Y State Dent J 2015; 81: 30–3. 26749781

[13] Rega AJ, Aziz SR, Ziccardi VB. Microbiology and antibiotic sensitivities of head and neck space infections of odontogenic origin. J Oral Maxillofac Surg 2006; 64: 1377–80. 16916672 10.1016/j.joms.2006.05.023

[14] Sousa EL, Gomes BP, Jacinto RC, Zaia AA, Ferraz CC Microbiological profile and antimicrobial susceptibility pattern of infected root canals associated with periapical abscesses. Eur J Clin Microbiol Infect Dis 2013; 32: 573–80. 23224675 10.1007/s10096-012-1777-5

[15] Warnke PH, Becker ST, Springer IN, Haerle F, Ullmann U, Russo PA, et al Penicillin compared with other advanced broad spectrum antibiotics regarding antibacterial activity against oral pathogens isolated from odontogenic abscesses. J Craniomaxillofac Surg 2008; 36: 462–7. 18760616 10.1016/j.jcms.2008.07.001

